# Correction to “Spatial and temporal independent component analysis of functional MRI data containing a pair of task‐related waveforms”

**DOI:** 10.1002/hbm.70007

**Published:** 2024-08-27

Calhoun, V.D. , Adali, T. , Pearlson, G.D. , & Pekar, J.J. (2001). Spatial and temporal independent component analysis of functional MRI data containing a pair of task‐related waveforms. *Human Brain Mapping*, *13*, 43–53. 10.1002/hbm.1024 PMC687195611284046

In the author affiliations section, the affiliation of T. Adali was incorrect. It was stated as “Department of CSEE, University of Maryland, Baltimore, Maryland.” This should have read: “Department of CSEE, University of Maryland Baltimore County, Maryland.”

We apologize for this error.

